# Virulence Pattern of *Pyricularia oryzae* Pathotypes Towards Blast Monogenic Lines

**DOI:** 10.21315/tlsr2021.32.3.8

**Published:** 2021-09-30

**Authors:** Siti Norsuha Misman, Mohd Shahril Firdaus Ab Razak, Nur Syahirah Ahmad Sobri, Latiffah Zakaria

**Affiliations:** 1 Paddy and Rice Research Centre MARDI Seberang Perai, 13200 Kepala Batas, Pulau Pinang, Malaysia; 2 Biotechnology and Nanotechnology Research Centre, MARDI Headquarters, 43000 Serdang, Selangor, Malaysia; 3 School of Biological Sciences, Universiti Sains Malaysia, 11800 USM Pulau Pinang, Malaysia

**Keywords:** *Pyricularia oryzae*, Rice Blast, Pathotypes, Virulence Patterns, *Pyricularia oryzae*, Karah Padi, Patotip, Corak Kevirulenan

## Abstract

Rice blast caused by *Pyricularia oryzae* (*P. oryzae*) is one of the most serious diseases infecting rice worldwide. In the present study, virulence pattern of six *P. oryzae* pathotypes (P0.0, P0.2, P1.0, P3.0, P7.0 and P9.0) identified from the blast pathogen collected in Peninsular Malaysia, were evaluated using a set of 22 IRRI-bred blast resistance lines (IRBL) as well as to determine the resistance genes involved. The information on the virulence of the blast pathotypes and the resistance genes involved is important for breeding of new rice variety for durable resistance against blast disease. The IRBL was established from 22 monogenic lines, harbouring 22 resistance genes [*Pia, Pib, Pii, Pit, Pi3, Pi5(t), Pish, Pi1, Pik, Pik-s, Pik-m, Pik-h, Pik-p, Pi7(t), Pi9, Piz, Piz-5, Piz-t, Pi19, Pi20(t), Pita-2*, and *Pita=Pi4(t)*]. Based on the disease severity patterns, the tested pathotypes were avirulence towards seven IRBLs [IRBLi-F5, IRBLk-Ka, IRBLkh-K3, IRBLz-Fu, IRBLsh-S, IRBLPi7 (t) and IRBL9-W] of which these IRBLs harbouring *Pii, Pik, Pik-h, Piz, Pish*, *Pi7(t)* and *Pi9* resistance genes, respectively. Therefore, the results suggested that the seven IRBLs carrying seven resistance genes [*Pii, Pik, Pik-h, Piz, Pish*, *Pi7(t)* and *Pi9*] would be suitable candidates of resistance genes to be incorporated in new breeding lines to combat the current blast pathotypes in the field.

## Abstract (fr)

Karah padi yang disebabkan oleh *Pyricularia oryzae* merupakan salah satu penyakit paling signifikan yang memberi kesan terhadap tanaman padi di seluruh dunia. Dalam kajian ini, corak kevirulenan enam patotip, P0.0, P0.2, P1.0, P3.0, P7.0 dan P9.0 yang dikenal pasti dari pencilan *P. oryzae* yang dikumpul dari Semenanjung Malaysia, dinilai menggunakan satu set 22 galur rintangan biakan karah-IRRI (IRBL) serta untuk menganggar gen rintangan yang terlibat. Maklumat mengenai kevirulenan patotaip karah dan gen rintangan terlibat adalah penting untuk pembiakbakaan varieti padi baru untuk kerintangan terhadap penyakit karah. IRBL dibangunkan dari 22 galur monogen, mengandungi 22 gen rintangan [*Pia, Pib, Pii, Pit, Pi3, Pi5(t), Pish, Pi1, Pik, Pik-s, Pik-m, Pik-h, Pik-p, Pi7(t), Pi9, Piz, Piz-5, Piz-t, Pi19, Pi20(t), Pita-2*, and *Pita=Pi4(t)*]. Berdasarkan corak tindakbalas penyakit, patotip yang diuji adalah avirulens terhadap tujuh IRBL [IRBLi-F5, IRBLk-Ka, IRBLkh-K3, IRBLz-Fu, IRBLsh-S, IRBLPi7(t) dan IRBL9-W] yang mengandungi gen rintangan *Pii, Pik, Pik-h, Piz, Pish, Pi7(t)* dan *Pi9*, masing-masing. Oleh itu, hasil kajian mencadangkan tujuh IRBL yang membawa tujuh gen rintangan [*Pii, Pik, Pik-h, Piz, Pish, Pi7(t)* dan *Pi9*] boleh menjadi calon gen rintangan yang sesuai untuk digabungkan ke dalam galur pembiakbakaan baharu untuk memerangi patotip karah di lapangan.

**Highlights**

- Virulence patterns of six pathotypes of *P. oryzae* (P0.0, P0.2, P1.0, P3.0, P7.0 and P9.0) in Peninsula Malaysia were evaluated using a set of 22 IRRI-bred blast resistance lines (IRBL) to determine the response of the resistance genes against specific lines.
- Based on disease severity patterns, the six pathotypes were avirulence towards seven IRBLs [IRBLi-F5, IRBLk-Ka, IRBLkh-K3, IRBLz-Fu, IRBLsh-S, IRBLPi7 (t) and IRBL9-W] of which these IRBLs harbouring *Pii*, *Pik*, *Pik-h*, *Piz*, *Pish*, *Pi7(t)* and *Pi9* resistance genes, respectively.
- To combat rice blast against current high frequencies of pathotypes P7.0, P0.0, P9.0 and P1.0, suitable resistance genes or donor to be incorporated for developing future rice blast resistant variety are *Pii*, *Pik*, *Pik-h*, *Piz*, *Pish*, *Pi7(t)* and *Pi9*.

## INTRODUCTION

Blast disease caused by *Pyricularia oryzae* (*P. oryzae*) (synonym *Magnaporthe oryzae*) is one of most serious disease of rice worldwide including Malaysia. The most effective and practical method to control blast disease is the use of resistant rice varieties. However, the resistance often breakdown or lost in a few years after the rice variety was released. This is caused by the appearance of new virulence pathotypes or races of the blast pathogen that overcome the resistance (Zhou *et al*. 2007) as well as due to high variability of rice blast pathogen (Wang *et al*. 2013; Poonsin & Parinthawong 2020). In order to develop more effective resistance against blast pathogen, it is important to have the knowledge of the host resistance and the pathogen as well as to determine the resistance genes.

The interaction between rice plant and blast pathogen is based on gene-for-gene theory by Flor (1971) of which for every resistance gene (*R* gene) in the host, there is a corresponding avirulence gene (*AVR*) in the pathogen. Thus, in blast disease pathosystem, a major resistance gene confers specific resistance to a blast pathogen pathotype that contains a specific corresponding avirulence gene. In other words, every resistance gene in the host corresponds to an avirulence gene in the pathogen that acts as an effector to triggers the defence response (Wang *et al*. 2008; Huang *et al*. 2014).

To study the resistance and avirulence genes interaction, differential system comprising rice differential varieties and blast pathogen pathotypes are often used. This system provide systematic method to characterise and to postulate the resistance genes as well as to determine the relationships between pathotypes and the resistance genes (Koide *et al*. 2011). The information can be used to strategise effective and lasting method to manage blast disease of rice.

Differential varieties distinguish pathotypes by their differential reactions to the blast pathogen. Current pathotype identification by using local differential varieties is useful to study the pathological diversity of *P. oryzae* population for pathotype identification and to characterise the blast isolates, but it is not sufficient to distinguish and characterise in detail blast pathotypes as the system lack the information on the genes involved in these local differential varieties. Thus, a set of international differential varieties harbouring resistance genes is frequently used to determine blast pathotypes virulence patterns.

A set of international blast differential varieties were developed by collaborative effort between International Rice Research Institute (IRRI) and Japan International Research Center for Agricultural Sciences (JIRCAS). The international blast differential varieties consisted of 23 monogenic lines of IRRI-bred blast-resistant line (IRBL) that representing 23 resistance genes, namely *Pish, Pia, Pib, Pit, Pii, Pi1, Pi3, Pi5(t), Pik, Pik-s, Pik-m, Pik-h, Pik-p, Pi7(t), Pi9, Piz, Piz-5, Piz-t, Pita-2, Pita, Pi12(t), Pi19(t)* and *Pi20(t)* with the genetic background of a Chinese Lijiang Xintuan Heigu (LTH) rice variety from blast-susceptible Japonica variety (Tsunematsu *et al*. 2000; Kobayashi *et al*. 2007). This differential system is used for identification of pathotype virulence, virulence pattern between the blast pathotypes and predicting the resistance gene(s) in rice varieties (Telebanco-Yanoria *et al*. 2010).

In Peninsular Malaysia, six pathotypes P0.0, P0.2, P1.0, P3.0, P7.0 and P9.0 of *P. oryzae* were identified from blast disease samples collected from 2014–2016 (Siti Norsuha & Latiffah 2019). Thus, the objectives of this study were to evaluate the virulence patterns of the blast pathotypes using international differential varieties and to predict the resistance genes corresponded to the blast pathotypes.

## MATERIALS AND METHODS

### Blast Monogenic Lines

In this study, a set of international differential varieties consisting of 22 IRBLs carrying 22 resistance genes [*Pia, Pib, Pii, Pit, Pi3, Pi5(t), Pish, Pi1, Pik, Pik-s, Pik-m, Pik-h, Pik-p, Pi7(t), Pi9, Piz, Piz-5, Piz-t, Pi19, Pi20(t), Pita-2* and *Pita=Pi4(t)* and a susceptible control, LTH (Table 1) were used to evaluate the virulence of six pathotypes identified in Peninsular Malaysia. The resistance genes present in each line was predicted based on their reaction patterns to the IRBL. Only 22 IRBL lines were used in the present study as the seeds of IRBL12-M did not germinate and thus was not included in the study.

Sowing of the seeds and inoculum preparation were synchronised. Seeds were pre-germinated and sown in a plastic tray (26 cm × 37 cm) with sieved top soil mixed with cow dung. The seedlings were fertilised with urea (150N kg/ha) 2–3 days before inoculation.

#### Inoculum preparation

*P. oryzae* pathotypes used in this study were P13-1.3 (P0.0), K3-1.1 (P0.2), K11-8.1 (P1.0), B10-2.3 (P3.0), K8-1.2 (P7.0) and P14-4.2 (P9.0). Culture of the six *P. oryzae* pathotypes were sub-cultured and grown on oatmeal agar (OMA) at room temperature for 14 days. The OMA was manually prepared and to prepare 1L of the medium, rolled oats (50 g), sucrose (5 g) and agar (16 g) were used and distilled water was added to made up 1L of the medium. The medium was sterilised at 121°C for 15–20 min.

The inoculum preparation was done according to Hayashi *et al*. (2009). Mycelia grown on OMA were scraped with spatula and the plates were left open in a tray covered with wrapping plastic. The plates were then placed under exposure of fluorescent light at 25°C ± 2°C for 4–7 days in order to induce sporulation. To prepare a conidial suspension, the plates were flooded with distilled water and the conidia were gently scraped using a brush. The conidial suspensions were filtered through nylon mesh and the concentrations of the suspensions were adjusted to 1×10^5^ spores/ml by using a haemocytometer.

#### Inoculation of P. oryzae pathotypes

Rice seedlings at 3–4 leaf stage were inoculated with different pathotypes of blast isolates by spraying the conidial suspension simultaneously using a motorised sprayer. The inoculated seedlings were placed in a dark chamber with a moisture-saturated atmosphere at 25°C–30°C for 24 hr. The seedlings were then transferred to a mist room with high humidity for 6 days (Hayashi *et al*. 2009). The seedlings were arranged in complete randomised design with three replicates in a plant house at MARDI Seberang Perai, Pulau Pinang, Malaysia. The inoculation and evaluation were repeated twice.

#### Disease assessment

Disease severity was assessed 7 days after inoculation using disease scale as described by Goto and Yamanaka (1968) and Mackill and Bonman (1992) (Table 2). Disease assessment scored as 0 to 2 is categorised as resistant (R) and scored 3 to 5 is categorised as susceptible (S) as described by Hayashi *et al*. (2009). Percentage of resistant or susceptible reaction was calculated as follows:


$$\text{Percentage of resistant or susceptible reaction}=\frac{\text{No}\text{. of IRBL showed resistant or susceptible reactions}}{\text{Total number of IRBL evaluated}}\times 100$$

#### Statistical analysis

SAS statistical software package version 9.4 (SAS Institute, Cary, NC) was used for statistical analysis. Disease score for virulence analysis was found to be non-normally distributed, therefore the data was analysed using nonparametric method: Kruskal-Wallis test (*p* ≤ 0.05).

Cluster analysis was conducted to classify the pathotypes based on the disease severity patterns. For cluster analysis, disease scores were converted to binary system where the resistant reaction was scored as 0 while the susceptible reaction was scored as 1. The binary data of the disease score was entered for analysis using NTSYS-pc 2.2 software (Rohlf 2005). Dendogram of cluster analysis was performed based on matrix of similarities between all pair of isolates (Jaccard coefficient) by Unweighted Pair Group Method with Arithmetic Mean (UPGMA). Jaccard coefficient was chosen as the coefficient measured similarity between all pair of isolates and negative matches are not counted (Romesburg 1984).

## RESULTS AND DISCUSSION

Disease severity pattern of IRBL to the six blast pathotypes identified in Peninsular Malaysia is shown in Table 3. Determination on the virulence pattern of the six pathotypes (P0.0, P0.2, P1.0, P3.0, P7.0 and P9.0) would help to reveal the response of the resistance genes against specific lineages by using a set of international differential variety.

Based on the reactions of 22 IRBL harbouring 22 resistance genes [*Pia, Pib, Pii, Pit, Pi3, Pi5(t), Pish, Pi1, Pik, Pik-s, Pik-m, Pik-h, Pik-p, Pi7(t), Pi9, Piz, Piz-5, Piz-t, Pi19, Pi20(t), Pita-2*, and *Pita=Pi4(t)*] and LTH, the results showed that seven IRBLs, IRBLi-F5, IRBLk-Ka, IRBLkh-K3, IRBLz-Fu, IRBLsh-S, IRBLPi7(t) and IRBL9-W, harbouring *Pii, Pik, Pik-h, Piz, Pish*, *Pi7(t)* and *Pi9*, respectively were resistant to all pathotypes evaluated.

Meanwhile, IRBL carrying resistance genes, *Pib* (IRBLb-B) and *Pit* (IRBLt-K59) were susceptible to all pathotypes tested. This suggested that the resistance genes are not suitable to be incorporated in developing new resistant variety.

Virulence of the pathotypes was determined by analysing the disease score scale using Kruskal-Wallis test. The analysis showed that the difference in median of disease score were significant among the six pathotypes (*p* ≤ 0.001) as shown in Table 4. Among the pathotypes evaluated, pathotype P7.0 was the most virulent based on the highest mean ranking value of 248.01. The second virulent pathotype based on the mean ranking value was P1.0 followed by pathotypes P0.0, P9.0 P0.2 and P3.0.

Based on the disease severity pattern, the number and percentage of IRBL showed resistant or susceptible reactions to the six blast pathotypes is shown in Table 5. The results indicated similar ranking patterns of virulence from Kruskal-Wallis test where pathotype P7.0 showed the highest virulence among the pathotypes with 40.9% of the IRBL were susceptible, followed by pathotypes P0.0 and P1.0 with the percentage of susceptible reaction of 36.4%. Both pathotypes P0.2 and P9.0 were considered as intermediate virulence to the IRBL tested with percentage of susceptible reaction of 31.8%. Subsequently, pathotype P3.0 was the least virulent pathotype where 81.8% of the IRBL were resistant.

The most virulent pathotype, P7.0 which also the dominant pathotype in rice field in Peninsular Malaysia (Siti Norsuha & Latiffah 2019) was avirulent to 13 IRBL, namely IRBLi-F5, IRBLk-Ka, IRBLkh-K3, IRBLz-Fu, IRBLta-K1, IRBLsh-S, IRBLP5-M, IRBLPi7(*t*), IRBL9-W, IRBL19-A, IRBLPik-m and IRBLta2-Re which harbour the resistance genes *Pii,Pik, Pik-p, Pik-h, Pita, Pish, Pi7(t), Pi9, Pi19, Pik-m* and *Pita-2*, respectively. In particular, IRBL carrying *Pii, Pik, Pik-h, Piz, Pish*, *Pi7(t)* and *Pi9* were observed to be resistant to all pathotypes evaluated. The least virulent pathotype, P3.0 was virulent to IRBLzt-T, IRBLb-B, IRBLt-K59 and IRBL3-C4, carrying resistance genes *Piz-t, Pib, Pit* and *Pi3*, respectively.

Pathotype P0.0 was avirulent to susceptible control variety, LTH. The results suggested that LTH may harboured the genes that confer specific resistance to pathotype P0.0. Similar results were reported by Fukuta *et al*. (2014) of which 3.3% of blast isolates tested in Cambodia showed avirulence to LTH, suggesting that the susceptible variety may contain resistance genes in its genetic background. However, according to Ling *et al*. (1995) the resistance genes in LTH were of minor importance, and Tsunematsu *et al*. (2000) reported major resistance genes have not been identified in LTH.

Based on the disease severity pattern, each pathotype was either virulent or avirulent on specific monogenic lines and the monogenic lines were also susceptible or resistant against specific pathotypes. Ellingboe and Chao (1994) described that the ability of a plant to express resistance depends on the genotype of the pathogen. Thus, the results from this study was an agreement with the gene-for-gene hypothesis by Flor (1971) of which the presence of major resistance gene (*R* gene) in the plant is effective in recognising avirulence gene (*AVR* gene) in *P. oryzae* pathotypes (Jones & Dangl 2006; Lu *et al*. 2019). Subsequently, the race-specific pathogen recognition will trigger the signal transduction events that lead to pathogen invasion and their virulence functions.

Results from this study shown the avirulence of all pathotypes evaluated to IRBL harbouring R genes, *Pii, Pik, Pik-h, Piz, Pish*, *Pi7(t)* and *Pi9*. The results suggested that the six pathotypes may contain the *AVR* gene, avr-*Pii*, avr-*Pik*, avr-*Pik-h*, avr-*Piz*, avr-*Pish*, avr-*Pi7(t)* and avr-*Pi9* that recognised *R* gene in their respective IRBL. Therefore, these *R* genes, namely *Pii, Pik, Pik-h, Piz, Pish*, *Pi7(t)* and *Pi9* could be suitable candidates of resistance genes to be incorporated in the new breeding lines in terms of combating the current pathotypes in the field.

**Figure 1:**
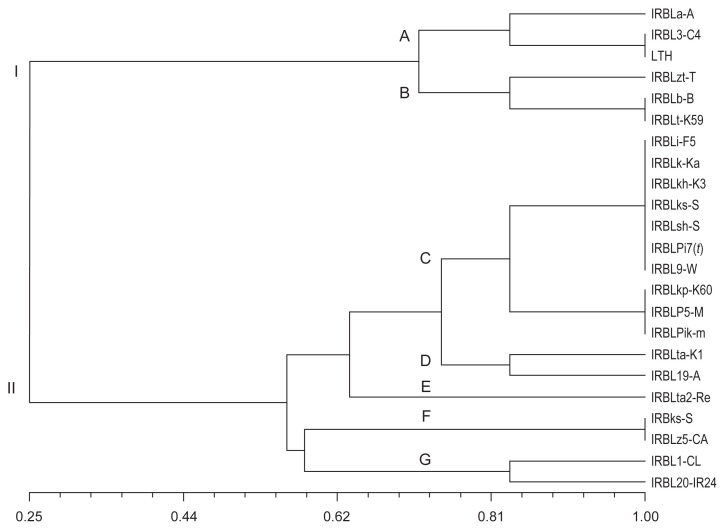
Cluster analysis using frequency of disease score of *P.oryzae* pathotypes against IRBL.

Cluster analysis carried out using binary data of disease score based on Jaccard similarity coefficient is presented in a dendrogram (Fig. 1). The cluster analysis suggested the disease score can be grouped into two main clusters, I and II at similarity coefficient of 0.25. Cluster I was divided into two sub-clusters, A and B, while cluster II comprising five sub-clusters, C, D, E, F and G.

Sub-cluster A consisted of IRBLs, IRBLa-A (*Pia*) and IRBL3-C4 (*Pi3*) as well as LTH which were resistant to pathotype P0.0. Sub-cluster B consisted of IRBLt-K59 (*Pit*) and IRBLb-B (*Pib*) which were susceptible to all pathotypes tested and IRBLzt-T (*Piz-t*) was resistant to only P0.2.

Sub-cluster C comprised IRBLi-F5 (*Pii*), IRBLk-Ka (*Pik*), IRBLkh-K3 (*Pik-h*), IRBLz-Fu (*Piz*), IRBLsh-S (Pish), IRBLPi-7(*t*) [*Pi7(t)*] and IRBL9-W (*Pi-9*) which were resistant to all pathotypes tested. Another group in this cluster includes IRBLkp-K60 (*Pik-p*), IRBLP5-M [*Pi5(t)*] and IRBLPik-m (*Pik-m*) of which these lines were susceptible to only pathotype P0.0 and resistant to the rest of the pathotypes evaluated.

Sub-cluster D included IRBLta-K1 (*Pita*) and IRBL19-A (*Pi19*), both susceptible to pathotype P1.0. Only IRBLta2-Re (*Pita2*) was grouped in sub-cluster E of which the line was susceptible to pathotypes P0.2 and P1.0. Both sub-clusters D and E were resistant to pathotypes P3.0, P7.0 and P9.0.

Sub-cluster F contained two lines, IRBLks-S (*Pik-s*) and IRBLz5-CA (*Piz-5*) which were susceptible to pathotypes P7.0 and P9.0. Two lines, RBL1-CL (*Pi1*) and IRBL20-IR24 [*Pi20(t)*] susceptible to pathotypes P0.2 and P7.0 were grouped in sub-cluster G.

Pathogenicity and virulence patterns of blast isolates in many countries have been identified using differential varieties with different types and numbers of resistance gene. The present study was similar with a study conducted by Khan *et al*. (2014) on the inoculation of blast isolates from fragrant rice in Bangladesh, on a set of international differential varieties consisting of 32 monogenic lines. In the study, *Pish, Pi9, Pita-2* and *Pita* were estimated as the effective resistance genes against tested blast isolates, and 80%–90% resistance frequencies were observed.

In Japan, pathogenicity study was carried out on 310 blast isolates from eight regions then challenged to a set of differential varieties consisted of 23 monogenic lines (carrying 21 resistance genes) and two near-isogenic line with the LTH genetic background. From the 310 blast isolates tested, 306 isolates (98.7%) were found to be virulent to IRBLsh-S (*Pish*) (Kawasaki-Tanaka & Fukuta 2014). In contrast, the results of the present study revealed that all the pathotypes tested were avirulent to IRBLsh-S (*Pish*).

The resistance gene *Pii* which could be one of suitable candidates of resistance genes to be used to develop new breeding lines, has been deployed for more than two decades in China (Duan *et al*. 1990). However, Zhu *et al*. (2000) and Li *et al*. (2007) reported the rice cultivars that harbored *Pii* gene were resistant to only 54.6% of blast isolates evaluated in Yunnan province and 15.1% isolates from Guangdong province, respectively. Subsequently, studies conducted by Huang *et al*. (2014) on blast isolates from various rice-producing regions in China revealed that 80% of the isolates were found to have complete deletions of *AVR-Pii*. The results suggested breakdown of *Pii* resistance in the rice cultivars. The *Pii* gene composed of five exons encoding a putative CC-NBS-LRR protein with 1025 amino acids (Takagi *et al*. 2013). The *AVR-Pii* gene of blast pathogen corresponds with the host resistance gene *Pii*, subsequently triggers the defence response (Yoshida *et al*. 2009).

Studies conducted by Silva *et al*. (2004) and Yasuda *et al*. (2006) revealed that a loss of the *AVR-Pii* gene transforms an avirulent to a virulent fungal strain. Moreover, the *AVR-Pii* gene is located on chromosome 7 which is a highly unstable chromosome segment (Yasuda *et al*. 2006; Yoshida *et al*. 2009) which suggested a risk of gene loss and horizontal transfer events (Silva *et al*. 2004; Rehmeyer *et al*. 2006).

Although in the present study, the blast isolates used were limited, determination on the virulence patterns of the six pathotypes of *P. oryzae* (P0.0, P0.2, P1.0, P3.0, P7.0 and P9.0) help to determine the response of the resistance genes against specific lines. Thus, it is an initial step to elucidate the diversity and differentiation of blast pathotypes virulence using differential varieties. The knowledge on the virulence of the blast pathotypes and the resistance genes is important for breeding of new rice variety for durable resistance against blast disease.

The variability in virulence patterns of the blast pathotypes would greatly help researchers in selecting suitable donors in breeding for resistance of rice blast of which particular functioning set of genes are incorporated into a desirable rice variety for durable resistance against the blast pathogen.

## CONCLUSION

In conclusion, to combat rice blast against current high frequencies of pathotypes P7.0, P0.0, P9.0 and P1.0, suitable resistance genes or donor to be incorporated for developing future rice blast resistant variety are *Pii, Pik, Pik-h, Piz, Pish*, *Pi7(t)* and *Pi9*. Meanwhile, *Pib* and *Pit* are not suitable resistance genes to be incorporated for developing new resistant variety considering their susceptible response to all pathotypes evaluated.

## Figures and Tables

**Table 1:** International differential varieties consisting of 22 IRRI-bred blast resistance lines (IRBL) and target resistant genes.

Designation of IRBLs	Target resistant gene
IRBLa-A	*Pia*
IRBLi-F5	*Pii*
IRBLks-S	*Pik-s*
IRBLk-Ka	*Pik*
IRBLkp-K60	*Pik-p*
IRBLkh-K3	*Pik-h*
IRBLz-Fu	*Piz*
IRBLz5-CA	*Piz-5*
IRBLzt-T	*Piz-t*
IRBLta-K1	*Pita=Pi4(t)*
IRBLb-B	*Pib*
IRBLt-K59	*Pit*
IRBLsh-S	*Pish*
IRBL1-CL	*Pi1*
IRBL3-C4	*Pi3*
IRBLP5-M	*Pi5(t)*
IRBLPi7(*t*)	*Pi7(t)*
IRBL9-W	*Pi9*
IRBL19-A	*Pi19*
IRBLPik-m	*Pik-m*
IRBL20-IR24	*Pi20(t)*
IRBLta2-Re	*Pita-2*

Other varieties evaluated	

Lijiang Xintuan Heigu (LTH)	Susceptible
MR211 (MARDI released varieties)	Susceptible check
MR84 (MARDI released varieties)	Resistant check

**Table 2:** Disease scale for blast disease assessment on IRBLs.

Scale	Blast symptom
0	No evidence of infection.
1	Brown specks smaller than 0.5 mm in diameter. No sporulation. Uniform or scattered brown specks.
2	Brown specks about 0.5 mm–1.0 mm in diameter. Small lesions with distinct tan centre which surrounded by a darker brown margin approximately 1 mm in diameter. No sporulation.
3	Roundish to elliptical lesion about 1 mm–3 mm in diameter with gray centre surrounded by brown margins. Small eyespot lesions less than one and a half times the interval between thin veins or less than 1.5 mm in diameter surrounded by dark brown, lesions capable of sporulation.
4	Typical spindle shaped blast lesion capable of sporulation, 3 mm or longer with necrotic gray centre and water soaked brown margins with little or no coalescence of lesion. Intermediate size eyespot lesions less than twice the size of interval between thin veins or less than 2 mm in diameter.
5	Lesions as in scale 4 but about half of one or two leaf blade killed by coalescence of lesion. Large eyespot lesions sized more than twice the size of interval between thin veins or more than 2 mm in diameter.

**Table 3:** Disease severity pattern of IRBL to six *P. oryzae* pathotypes identified in Peninsular Malaysia.

Designation of IRBL	Resistance Gene	P0.0	P0.2	P1.0	P3.0	P7.0	P9.0
IRBLa-A	*Pia*	R	S	S	R	S	S
IRBLi-F5	*Pii*	R	R	R	R	R	R
IRBLks-S	*Pik-s*	R	R	R	R	S	S
IRBLk-Ka	*Pik*	R	R	R	R	R	R
IRBLkp-K60	*Pik-p*	S	R	R	R	R	R
IRBLkh-K3	*Pik-h*	R	R	R	R	R	R
IRBLz-Fu	*Piz*	R	R	R	R	R	R
IRBLz5-CA	*Piz-5*	R	R	R	R	S	S
IRBLzt-T	*Piz-t*	S	R	S	S	S	S
IRBLta-K1	*Pita=Pi4(t)*	R	R	S	R	R	R
IRBLb-B	*Pib*	S	S	S	S	S	S
IRBLt-K59	*Pit*	S	S	S	S	S	S
IRBLsh-S	*Pish*	R	R	R	R	R	R
IRBL1-CL	*Pi1*	R	S	R	R	S	R
IRBL3-C4	*Pi3*	R	S	S	S	S	S
IRBLP5-M	*Pi5(t)*	S	R	R	R	R	R
IRBLPi7(*t*)	*Pi7(t)*	R	R	R	R	R	R
IRBL9-W	*Pi9*	R	R	R	R	R	R
IRBL19-A	*Pi19*	S	R	S	R	R	R
IRBLPik-m	*Pik-m*	S	R	R	R	R	R
IRBL20-IR24	*Pi20(t)*	S	S	R	R	S	R
IRBLta2-Re	*Pita-2*	R	S	S	R	R	R
Lijiang Xintuan Heigu (LTH)	Susceptible	R	S	S	S	S	S
MR 211	–	S	R	S	R	S	S
MR 84	–	R	R	R	R	R	R

*Note*: R = Resistant, S = Susceptible, ‘-‘ = information on the resistance gene is not available.

**Table 4:** Disease score based on disease severity of IRBL against six pathotypes of *P. oryzae*.

Pathotype	*N*	Median	Mean rank
P7.0	74	2.00	248.01
P1.0	75	1.70	245.87
P0.0	75	2.00	233.93
P9.0	75	1.60	226.39
P0.2	74	1.20	220.41
P3.0	75	0.40	191.22

*Note*: Chi-square, H = 17.33 with df = 5 at *p* ≤ 0.05; Disease score = 0 to 5.

**Table 5:** Number and percentage of IRBL showed resistant or susceptible reactions to six *P. oryzae* pathotypes.

Pathotype	Resistant		Susceptible	
	
	Number	%	Number	%
P0.0	14	63.6	8	36.4
P0.2	15	68.2	7	31.8
P1.0	14	63.6	8	36.4
P3.0	18	81.8	4	18.2
P7.0	13	59.1	9	40.9
P9.0	15	68.2	7	31.8
